# Perioperative antihypertensive medication use is associated with no antifibrotic benefit but higher complications and revision rates following shoulder arthroplasty

**DOI:** 10.1007/s00590-026-04883-y

**Published:** 2026-08-01

**Authors:** Tarishi Parmar, Miguel Fiandeiro, Mohammad Daher, Akin Adio, Jad Lawand, Adam Khan, Joseph Abboud

**Affiliations:** 1 Division of Shoulder and Elbow Surgery, Rothman Orthopaedics, Philadelphia, USA; 2 Perelman School of Medicine, University of Pennsylvania, Philadelphia, USA; 3 John Sealy School of Medicine, The University of Texas Medical Branch at Galveston, Galveston, USA; 4 Department of Orthopedic Surgery, Southern California Permanente Medical Group, Panorama City, USA

**Keywords:** Total shoulder arthroplasty, Renin-angiotensin-aldosterone system inhibitors, Angiotensin-converting enzyme inhibitors, Angiotensin receptor blockers, Postoperative complications, Fibrosis

## Abstract

**Purpose:**

Renin-angiotensin-aldosterone system (RAAS) inhibitors, including angiotensin-converting enzyme inhibitors (ACEis) and angiotensin receptor blockers (ARBs), are commonly prescribed antihypertensive medications with proposed antifibrotic and bone-protective effects. Their impact on outcomes following total shoulder arthroplasty (TSA) remains unclear. This study evaluates the association between perioperative RAAS inhibitor exposure and postoperative outcomes after TSA.

**Methods:**

A retrospective cohort study using the TriNetX network identified patients undergoing primary shoulder arthroplasty (2005–2025), stratified by ACEi or ARB use. One-to-one propensity score matching was performed. Outcomes included 90-day medical complications and 1-year mechanical complications and revision.

**Results:**

Post-matching, 27,938 patients per cohort (ARB vs. control) and 30,602 per cohort (ACEi vs. control) were included. Neither ARB nor ACEi use reduced rates of manipulation under anesthesia or capsular release at one year. Both cohorts had higher 90-day rates of emergency department visits, readmission, myocardial infarction, and acute renal failure. ACEi use was additionally associated with pulmonary embolism, deep-vein thrombosis, transfusion, and sepsis. At one year, both cohorts showed higher rates of periprosthetic joint infection, aseptic loosening, and revision. Dislocation risk was increased with ACEi use only.

**Conclusion:**

Perioperative RAAS inhibitor use was not associated lower rates of antifibrotic procedures following TSA. Instead, RAAS inhibitor use was associated with higher rates of several perioperative medical complications and longer-term surgical events, including infection and revision. ACE inhibitor exposure demonstrated a broader complication profile compared with ARB exposure. However, because patients receiving RAAS inhibitors often have greater comorbidity burden, these findings should be interpreted as associative rather than causal.

**Level of evidence III:**

Retrospective Cohort Study.

**Supplementary Information:**

The online version contains supplementary material available at 10.1007/s00590-026-04883-y.

## Introduction

 The incidence of primary total shoulder arthroplasty (TSA) has increased substantially over the past decade, rising by more than 100% between 2011 and 2017 [1]. Hypertension is highly prevalent among patients undergoing shoulder arthroplasty, affecting an estimated 55–76% of this population. Angiotensin-converting enzyme inhibitors (ACEIs) and angiotensin receptor blockers (ARBs) are widely prescribed first-line antihypertensive medications that act through inhibition of the renin–angiotensin–aldosterone system (RAAS) [2].

Perioperative management of RAAS inhibitors remains controversial. Continuation of ACEIs and ARBs has been associated with increased intraoperative hypotension, although randomized trials have not demonstrated differences in mortality or major adverse cardiovascular events between continuation and discontinuation strategies [3]. Beyond their cardiovascular effects, RAAS inhibitors exhibit antifibrotic properties that have gained increasing attention in orthopaedic surgery. Angiotensin II promotes fibroblast proliferation, myofibroblast differentiation, and collagen deposition via transforming growth factor–β (TGF-β) signaling, and inhibition of this pathway attenuates pathologic scar formation [4]. Given the role of fibrosis in postoperative stiffness, pain, and implant-related failure, these effects may be clinically relevant in arthroplasty populations. In total knee arthroplasty, postoperative ACEI or ARB use has been associated with lower rates of manipulation under anesthesia, arthroscopic lysis of adhesions, aseptic loosening, periprosthetic fracture, and revision surgery [5–7].

Despite the high prevalence of RAAS inhibitor use among patients undergoing shoulder arthroplasty, their association with postoperative complications and revision risk in this population remains poorly defined. Given the unique biomechanics of the shoulder and the importance of capsular healing and soft-tissue balance to functional outcomes, the potential influence of RAAS inhibition on postoperative recovery warrants further investigation. The purpose of this study is to evaluate the association between perioperative ACEI and ARB use and postoperative complications and revision rates following TSA.

## Methods

### Data source

This retrospective observational study utilized data from the TriNetX Research Network, a federated, Health Insurance Portability and Accountability Act (HIPPA)-compliant electronic health record (HER) platform. It includes more than 400 million patients across over 170 healthcare organizations in 30 countries. It captures longitudinal clinical information derived from both structured and unstructured EHR fields, including patient demographics, medication prescriptions, laboratory results, diagnostic codes mapped to the International Classification of Diseases, Tenth Revision (ICD-10), and procedural codes mapped to Current Procedural Terminology (CPT). To ensure cross-institutional consistency, all contributing sites harmonize data using a standardized common data model implemented by TriNetX. Patient records are fully de-identified and analyzed only in aggregate form, with no exchange of protected health information. Each individual is assigned a unique encrypted identifier to permit longitudinal tracking. In accordance with these safeguards, this study was deemed exempt from institutional review board oversight, consistent with TriNetX’s waiver from the Western Institutional Review Board.

### Cohort selection

Adult patients who underwent primary TSA between 2005 and 2025 were identified through relevant procedural coding, with TriNetX queried on December 10, 2025. Patients were stratified based on perioperative antihypertensive medication exposure. To isolate primary arthroplasty cases, patients were excluded if they had a history of prior shoulder arthroplasty or revision surgery involving the operative shoulder, a diagnosis of upper-extremity malignancy, or major traumatic injury requiring concurrent reconstructive procedures. Additionally, individuals without a minimum of two year of postoperative follow-up were excluded from the final cohort.

### Study population and propensity matching

In the first analysis, patients with documented angiotensin receptor blocker (ARB) use from one year prior to surgery through three months postoperatively were compared with patients without ARB exposure during this interval. In the second analysis, patients with documented angiotensin-converting enzyme inhibitor (ACEi) use during the same perioperative window were compared with patients without ACEi exposure. To minimize confounding from baseline differences, a propensity score–based matching approach was employed. Propensity scores were generated using multivariable logistic regression models that included patient demographics and relevant comorbidities detailed in Table 1. Patients were subsequently matched in a 1:1 ratio using a greedy nearest-neighbor algorithm with a caliper of 0.1 standard deviations of the pooled propensity score. Following matching, cohort balance was reassessed to confirm adequate equivalence before proceeding with postoperative outcome analyses.

**Table 1:** Characteristics of cohorts before and after propensity score matching: ARBs vs. controls

Characteristics	Cohort	Before matching			After matching		
		Patients	% patients	*P* value	Patients	% patients	*P* value
*Demographics*							
Age at index	ARBs Control	28,411 (71.4 ± 9.11) 95,537 (67.5 ± 11.4)	100.00 100.00	< 0.001	27,938 (71.3 ± 9.09) 27,938 (71.6 ± 9.19)	100.00 100.00	**< 0.001**
White	ARBs Control	24,050 82,894	84.65 86.77	< 0.001	23,720 23,783	84.90 85.13	0.455
Female	ARBs Control	16,737 52,525	58.91 55.00	< 0.001	16,418 16,430	58.77 58.81	0.918
Not Hispanic or Latino	ARBs Control	22,570 74,467	79.44 77.95	< 0.001	22,193 22,231	79.44 79.57	0.690
Hispanic or Latino	ARBs Control	803 2,667	2.83 2.79	0.7550	781 760	2.79 2.72	0.587
Black or African American	ARBs Control	2,669 6,709	9.39 7.02	< 0.001	2,565 2,468	9.18 8.83	0.152
Male	ARBs Control	11,670 42,968	41.08 44.97	< 0.001	11,516 11,497	41.22 41.15	0.870
Asian	ARBs Control	367 833	1.29 0.87	< 0.001	350 327	1.25 1.17	0.374
*Diagnosis*							
Diabetes mellitus	ARBs Control	9,888 18,146	34.80 18.99	< 0.001	9,512 9,301	34.05 33.29	0.060
Chronic kidney disease (CKD)	ARBs Control	5,103 7,495	20.12 8.78	< 0.001	4,709 4,549	19.01 18.36	0.065
Heart failure	ARBs Control	5,373 6,713	18.91 7.03	< 0.001	4,944 4,881	17.70 17.47	0.484
Hypertensive diseases	ARBs Control	25,004 50,679	88.01 53.05	< 0.001	24,531 24,543	87.80 87.85	0.877
Other diseases of liver	ARBs Control	3,107 6,151	10.94 6.44	< 0.001	2,924 2,869	10.47 10.27	0.478
Other rheumatoid arthritis	ARBs Control	1,987 4,716	7.83 5.53	< 0.001	1,893 1,806	7.64 7.29	0.137
Other diseases of arteries and arterioles	ARBs Control	2,122 3,351	7.47 3.51	< 0.001	1,929 1,904	6.91 6.82	0.070
Diseases of capillaries	ARBs Control	516 1,179	1.82 1.23	< 0.001	492 482	1.76 1.73	0.779
Other hyperlipidemia	ARBs Control	5,440 11,343	19.15 11.87	< 0.001	5,258 5,133	18.82 18.37	0.210
Obesity due to excess calories	ARBs Control	5,500 9,418	19.36 9.86	< 0.001	5,160 5,084	18.47 18.20	0.447
Other obesity	ARBs Control	2,561 3,971	9.01 4.16	< 0.001	2,349 2,288	8.41 8.19	0.395
Ischemic heart disease	ARBs Control	9,774 16,721	34.40 17.50	< 0.001	9,197 8,972	32.92 32.11	0.061
*Laboratory*							
Hemoglobin A1c	ARBs Control	11,854 (6.2 ± 1.12) 26,396 (6.02 ± 1.05)	53.24 33.82	< 0.001	11,493 (6.19 ± 1.11) 10,963 (6.16 ± 1.08)	52.53 50.11	0.120

Bold value indicates statistically significant differences (p < 0.05)

### Outcomes

The outcomes of interest included short-term medical and postoperative complications as well as longer-term surgical sequelae following TSA. Ninety-day outcomes encompassed emergency department visits, hospital readmissions, myocardial infarction, pulmonary embolism, deep vein thrombosis, blood transfusion, acute renal failure, and sepsis. Longer-term outcomes assessed within one year of surgery included subsequent capsular release, manipulation under anesthesia, revision arthroplasty, periprosthetic joint infection, implant loosening, and postoperative dislocation. All outcomes were evaluated within their predefined postoperative time intervals, and events occurring in fewer than ten patients were reported as “≤10” in accordance with TriNetX privacy requirements.

### Statistical analysis

Statistical analyses were conducted within the TriNetX Analytics environment, which supports data processing and computation using Java-, R-, and Python-based frameworks. Comparisons of categorical postoperative outcomes between cohorts were expressed as odds ratios with corresponding 95% confidence intervals, with group differences assessed using chi-square or Fisher’s exact tests as appropriate based on expected cell counts. A two-tailed P value of less than 0.05 was considered statistically significant. All outcome analyses were performed after propensity score matching to account for baseline differences between cohorts.

## Results

### ARBs vs. controls

#### Overall cohort demographics

Before matching, the ARB cohort included 28,411 patients and the control cohort included 95,537 patients. After 1:1 propensity score matching, 27,938 patients remained in each group.

Following matching, most demographic and clinical characteristics were well balanced between the cohorts. Patients receiving ARBs were slightly younger than controls (71.3 ± 9.09 years vs. 71.6 ± 9.19 years, *P* < 0.001). No significant differences remained between cohorts with respect to sex, race, ethnicity, or major comorbid conditions including diabetes mellitus, chronic kidney disease, heart failure, hypertensive disease, ischemic heart disease, liver disease, rheumatoid arthritis, vascular disease, hyperlipidemia, or obesity. Hemoglobin A1c levels were also comparable between groups after matching.

#### Ninety-day and one-year outcomes

Patients on ARBs experienced higher rates of some ninety-day complications after shoulder arthroplasty than patients not on ARBs (Table 2). Rates of emergency department (ED) visits (17.8% vs. 17.1%, OR 1.05, *P* = 0.035), readmission (15.4% vs. 13.3%, OR 1.18, *P* < 0.001), myocardial infraction (MI) (2.6% vs. 1.6%, OR 1.66, *P* < 0.001) and renal failure (6.5% vs. 5.4%, OR 1.23, *P* < 0.001) were higher in the depression cohort. Other ninety-day complications, including pulmonary embolism (PE), deep vein thrombosis (DVT), transfusion and sepsis, did not differ significantly between groups.

**Table 2:** Comparison of postoperative outcomes between ARB users and matched controls

Outcome	Incidence (%)		Odds ratio	95% confidence interval	*P* value
	ARBs	Control			
*90-days*					
ED visits	17.85%	17.13%	1.05	1.00-1.10	**0.035**
Readmission	15.37%	13.34%	1.18	1.10–1.20	**< 0.001**
MI	2.56%	1.56%	1.66	1.46–1.89	**< 0.001**
PE	1.47%	1.28%	1.15	0.99–1.34	0.064
DVT	2.33%	2.31%	1.01	0.90–1.13	0.905
Transfusion	1.70%	1.68%	1.02	0.88–1.16	0.834
Renal failure	6.55%	5.405%	1.23	1.14–1.32	**< 0.001**
Sepsis	1.89%	1.90%	1.00	0.87–1.13	0.948
*1-year*					
Capsular release	0.1%	0.1%	0.94	0.47–1.863	0.862
MUA	0.0%	0.1%	0.53	0.24–1.13	0.095
Revision	2.7%	2.0%	1.40	1.24–1.59	**< 0.001**
PJI	2.9%	2.2%	1.306	1.16–1.47	**< 0.001**
Loosening	1.6%	1.2%	1.35	1.15–1.58	**< 0.001**
Dislocation	2.4%	2.2%	1.101	0.97–1.25	0.128

Bold values indicate statistically significant differences (p < 0.05)

**Table 3:** Characteristics of cohorts before and after propensity score matching: ACEis vs. controls

Characteristics	Cohort	Before matching			After matching		
		Patients	% patients	*P* value	Patients	% patients	*P* value
*Demographics*							
Age at index	ACEis Control	30,664 (69.5 ± 9.7) 93,295 (68 ± 11.3)	100.00 100.00	< 0.001	30,602 (69.5 ± 9.7) 30,602 (69.9 ± 9.76)	100.00 100.00	**< 0.001**
White	ACEis Control	26,710 80,242	87.10 86.01	< 0.001	26,652 26,738	87.09 87.37	0.298
Female	ACEis Control	15,604 53,659	50.89 57.51	< 0.001	15,601 15,350	50.98 50.16	**0.042**
Not Hispanic or Latino	ACEis Control	24,780 72,262	80.81 77.45	< 0.001	24,727 24,780	80.80 80.97	0.586
Hispanic or Latino	ACEis Control	906 2,565	2.95 2.75	0.0587	902 866	2.95 2.83	0.385
Black or African American	ACEis Control	2,319 7,061	7.56 7.57	0.9732	2,318 2,207	7.57 7.21	0.086
Male	ACEis Control	15,050 39,598	49.08 42.44	< 0.001	14,992 15,239	48.99 49.80	**0.046**
Asian	ACEis Control	162 1,037	0.53 1.11	< 0.001	162 180	0.53 0.59	0.329
*Diagnosis*							
Diabetes mellitus	ACEis Control	10,649 17,301	34.73 18.54	< 0.001	10,588 10,373	34.60 33.90	0.067
Chronic kidney disease (CKD)	ACEis Control	4,912 9,154	16.02 9.81	< 0.001	4,902 4,785	16.02 15.64	0.195
Heart failure	ACEis Control	4,578 6,999	14.93 7.502	< 0.001	4,539 4,452	14.83 14.55	0.320
Hypertensive diseases	ACEis Control	26,434 49,329	86.20 52.87	< 0.001	26,372 26,388	86.18 86.23	0.851
Other diseases of liver	ACEis Control	2,860 6,220	9.33 6.67	< 0.001	2,855 2,717	9.33 8.88	0.073
Other disorders of arteries and arterioles	ACEis Control	1,832 3,465	5.98 3.71	< 0.001	1,829 1,779	5.98 5.81	0.425
Diseases of capillaries	ACEis Control	414 1,235	1.35 1.32	< 0.001	414 380	1.35 1.24	0.263
Other hyperlipidemia	ACEis Control	5,610 11,143	18.30 11.94	< 0.001	5,597 5,397	18.29 17.64	0.052
Obesity due to excess calories	ACEis Control	5,023 9,571	16.38 10.26	< 0.001	5,011 4,819	16.38 15.75	0.051
Other obesity	ACEis Control	2,076 4,204	6.77 4.51	< 0.001	2,072 1,994	6.77 6.52	0.240
Ischemic heart disease	ACEis control	9,454 16,985	30.83 18.21	< 0.001	9,408 9,232	30.74 30.17	0.122
*Laboratory*							
Hemoglobin A1c	ACEis Control	15,484 (6.22 ± 1.32) 31,940 (5.99 ± 1.08)	50.50 34.24	< 0.001	15,447 (6.22 ± 1.32) 14,923 (6.19 ± 1.17)	50.48 48.77	0.050

Bold values indicate statistically significant differences (p < 0.05)

At one year, rates of revision (2.7% vs. 2.0%, OR 1.40, *P* < 0.001), peri-prosthetic infection (PJI) (2.9% vs. 2.2%, OR 1.31, *P* < 0.001) and loosening (1.6% vs. 1.2%, OR 1.35, *P* < 0.001) were higher among patients on ARBs.

Persistent elbow pain was more common among patients with depression than those without (35.8% vs. 32.6%, OR 1.15, *P* = 0.020). Capsular release, manipulation under anesthesia (MUA) and dislocation rates showed no significant differences between cohorts.

### ACEis vs. controls

#### Overall cohort demographics

Before matching, the ACE inhibitor cohort included 30,664 patients and the control cohort included 93,295 patients. After 1:1 propensity score matching, 30,602 patients remained in each group.

Following matching, most baseline characteristics were balanced between cohorts. Patients receiving ACE inhibitors were slightly younger than controls (69.5 ± 9.7 years vs. 69.9 ± 9.76 years, *P* < 0.001). Small differences in sex distribution remained, with a slightly lower proportion of females in the ACE inhibitor cohort compared with controls (50.98% vs. 50.16%, *P* = 0.042), and a corresponding difference in male distribution (48.99% vs. 49.80%, *P* = 0.046). No significant differences remained between groups with respect to race, ethnicity, diabetes mellitus, chronic kidney disease, heart failure, hypertensive disease, ischemic heart disease, liver disease, vascular disorders, hyperlipidemia, or obesity. Hemoglobin A1c levels were similar between cohorts after matching (6.22 ± 1.32 vs. 6.19 ± 1.17, *P* = 0.050).

#### Ninety-day and one-year outcomes

All the ninety-day complications after TSA were significantly higher for the patients on ACEi than the control (Table 4). Rates of ED visits (17.6% vs. 16.3%, OR 1.1, *P* < 0.001), readmission (15.7% vs. 12.1%, OR 1.35, *P* < 0.001), MI (2.6% vs. 1.4%, OR 1.86, *P* < 0.001), PE (1.5% vs. 1.1%, OR 1.31, *P* < 0.001), DVT (2.5% vs. 2.0%, OR 1.86, *P* < 0.001), transfusion (1.8% vs. 1.5%, OR 1.2, *P* = 0.007), renal failure (6.5% vs. 4.7%, OR 1.4, *P* < 0.001) and sepsis (2.2% vs. 1.6%, OR 1.3, *P* < 0.001) were all higher in the ACEi cohort.

**Table 4:** Comparison of postoperative outcomes between ACEi users and matched controls

Outcome	Incidence (%)		Odds ratio	95% confidence interval	*P* value
	ACEis	Control			
*90-days*					
ED visits	17.56%	16.28%	1.10	1.05–1.15	**< 0.001**
Readmission	15.73%	12.12%	1.35	1.29–1.42	**< 0.001**
MI	2.58%	1.40%	1.86	1.64–2.11	**< 0.001**
PE	1.49%	1.14%	1.31	1.13–1.52	**< 0.001**
DVT	2.54%	2.05%	1.24	1.11–1.39	**< 0.001**
Transfusion	1.76%	1.47%	1.20	1.05–1.38	**0.007**
Renal failure	6.47%	4.69%	1.40	1.30–1.51	**< 0.001**
Sepsis	2.18%	1.62%	1.35	1.19–1.53	**< 0.001**
*1-year*					
Capsular release	0.09%	0.06%	1.45	0.79–2.64	0.228
MUA	0.07%	0.09%	0.81	0.45–1.44	0.466
Revision	2.61%	2.08%	1.26	1.13–1.40	**< 0.001**
PJI	3.10%	2.36%	1.32	1.20–1.46	**< 0.001**
Loosening	1.48%	1.20%	1.24	1.07–1.42	**0.003**
Dislocation	2.67%	2.25%	1.20	1.08–1.33	**0.001**

Bold values indicate statistically significant differences (p < 0.05)

At one year, rates of revision (2.6% vs. 2.1%, OR 1.26, *P* < 0.001), PJI (3.1% vs. 2.4%, OR 1.32, *P* < 0.001) loosening (1.5% vs. 1.2%, OR 1.24, *P* < 0.003) and dislocation (2.7% vs. 2.2%, OR 1.20, *P* < 0.001) were higher among patients on ACEis. Capsular release and MUA showed no significant differences between cohorts.

## Discussion

In this large, matched analysis of patients undergoing TSA, preoperative exposure to renin-angiotensin-aldosterone system (RAAS) inhibitors, including angiotensin receptor blockers (ARBs) and angiotensin-converting enzyme inhibitors (ACEis), was statistically associated with higher observed rates of several 90-day and 1-year complications. Neither group demonstrated lower rates of subsequent antifibrotic procedures such as manipulation under anesthesia or capsular release. Both cohorts demonstrated higher observed rates of 90-day emergency department use, readmission, myocardial infarction, and acute renal failure. ACEi exposure was additionally associated with higher recorded rates of pulmonary embolism, deep-vein thrombosis, transfusion, and sepsis, a pattern that was not observed in the ARB cohort. At one year, both medication cohorts demonstrated higher rates of loosening, prosthetic joint infection (PJI), and revision procedures, while ACEi exposure was also associated with higher dislocation rates.

Studies have suggested that RAAS inhibitors may reduce postoperative stiffness through proposed antifibrotic effects [7–9]. Some shoulder studies specifically report that ARBs may lower rates of adhesive capsulitis and subsequent capsular release [8, 9]. However, in the present analysis we did not observe lower rates of manipulation under anesthesia or capsular release after shoulder arthroplasty among ACEi or ARB users. These findings are consistent with prior reports suggesting that the antifibrotic effects of RAAS blockade may not translate uniformly to postoperative arthrofibrosis outcomes [10–12]. Although a systematic review noted possible reductions in MUA and revision after TKA with perioperative ARB use, it emphasized the limited and heterogeneous nature of this evidence [13]. Miltenberg et al. further noted that large database studies have previously demonstrated protective effects of these antihypertensive agents against arthrofibrosis, whereas smaller studies do not [10]. Despite using a similarly large national database of 187,900 patients, we still found no antifibrotic signal, challenging the assumption that larger datasets inherently reveal benefit. Overall, the variability across joints, procedures, and study designs suggests that the relationship between RAAS inhibition and postoperative arthrofibrosis remains uncertain and warrants further investigation.

Interestingly, we observed higher rates of aseptic loosening in both ACEi and ARB cohorts, with ACEi users also demonstrating higher dislocation rates. Mechanistic studies show that RAAS activation promotes osteoclastogenesis, inflammatory bone loss, and high-turnover osteoporosis, while RAAS blockade suppresses these processes and is therefore theoretically bone-protective [14, 15]. Epidemiologic work similarly reports lower future osteoporotic fracture risk in hypertensive patients on RAAS inhibitors [16]. Albright et al. also found decreased aseptic loosening and revision rates among post-TKA ARB users [5]. These prior observations might suggest potential protective effects on periprosthetic bone integrity, which contrasts with the associations observed in the present analysis. One possible explanation is residual confounding related to the underlying comorbidity burden among patients prescribed these medications, which may persist despite propensity score matching. Additionally, the mechanical demands and biomechanics of shoulder arthroplasty differ substantially from lower-extremity arthroplasty, and the relationship between RAAS inhibition and mechanical outcomes in shoulder arthroplasty remains largely unexplored.

We also observed higher recorded rates of prosthetic joint infection in both medication cohorts. Prior work shows that ACE activity in myeloid cells is essential for antibacterial defense by supporting oxidative metabolism and ATP generation [17]. Its overexpression enhances resistance to Listeria and MRSA, whereas inhibition impairs anti-staphylococcal function in implant-infection models [18, 19]. Clinically, ACE inhibitor use has also been associated with higher PJI rates after TKA in some observational studies [20]. Furthermore, although ARBs do not directly inhibit myeloid ACE activity, we observed similar infection associations in both cohorts, suggesting that other patient-level factors or unmeasured confounders may contribute [20]. As such, these findings should be interpreted as associative observations rather than evidence of a causal immunologic effect of RAAS blockade.

The higher rates of myocardial infarction and acute renal failure observed in both medication cohorts may also reflect underlying cardiovascular risk profiles among patients requiring RAAS inhibition. ACEis and ARBs reduce angiotensin II-mediated vasoconstriction and can influence perioperative hemodynamics, and several studies have reported increased susceptibility to intraoperative hypotension in patients receiving these medications [21, 22]. However, the present dataset does not contain information regarding perioperative medication continuation, or hemodynamic parameters, limiting the ability to determine whether these events relate to pharmacologic effects.

Similarly, ACEi users demonstrated higher observed rates of sepsis and transfusion. ACE inhibition increases bradykinin signaling through reduced degradation, which may influence vascular tone and endothelial permeability [23]. Experimental work has suggested that bradykinin pathways may affect inflammatory and microvascular responses [23, 24]. While these mechanisms have been proposed in prior literature, they were not measured in the present dataset, and therefore cannot be directly evaluated in this analysis. Difference in baseline comorbidity burden is an alternative explanation.

Trikha et al. reported that perioperative ACEi use, but not ARB use, was associated with increased deep venous thrombosis and pulmonary embolism within 90 days [20]. Our findings similarly demonstrated higher thromboembolic event rates among ACEi users. Prior mechanistic work has suggested that RAAS inhibition, particularly ARBs, may exert endothelial-protective and antithrombotic effects [25–27]. Although a protective association for ARBs was not observed in the present study, the comparatively broader complication profile seen in the ACEi cohort may warrant further investigation in future studies.

Overall, these results underscore the need for careful preoperative assessment and coordination with medical teams when managing patients on chronic RAAS blockade. Future prospective studies with detailed clinical data, including perioperative medication management and cardiovascular risk stratification, will be necessary to clarify whether these associations reflect medication effects, patient comorbidity burden, or both.

### Limitations

Several limitations should be considered when interpreting the findings of this study. This analysis is based on retrospective data derived from a federated electronic health record network and is therefore subject to the inherent constraints of administrative coding. Diagnoses, complications, and revision procedures were identified using ICD and CPT codes, which may vary across institutions and providers. Misclassification of outcomes remains possible and could influence reported incidence rates. Medication exposure was identified based on documented prescriptions for ACE inhibitors or ARBs but could not be verified for perioperative continuation, dosage, duration of therapy, or patient adherence. The study also lacks granularity regarding surgical technique, implant selection, surgeon volume, and perioperative management strategies. These factors may independently influence complication rates and revision risk but are not captured within the dataset. Functional outcomes, pain scores, patient satisfaction, and quality-of-life measures were also unavailable, limiting insight into the clinical impact of the observed complications. Further, the retrospective observational design precludes causal inference. Additionally, residual confounding and selection bias are likely, as patients prescribed ACE inhibitors or ARBs may have a higher underlying comorbidity burden. Thus, despite propensity score matching, unmeasured factors such as disease severity, cardiovascular health, and frailty may influence outcomes.

## Conclusion

Perioperative RAAS inhibitor exposure was not associated with lower observed rates of postoperative antifibrotic procedures following total shoulder arthroplasty in this large database analysis. Instead, RAAS inhibitor use was associated with higher recorded rates of several perioperative medical complications and longer-term surgical events, including infection and revision. ACE inhibitor exposure demonstrated a broader complication profile compared with ARB exposure, However, since patients receiving RAAS inhibitors often have greater comorbidity burden that may persist despite propensity score matching, these results should be interpreted as associative rather than causal. Nevertheless, these findings highlight the importance of further investigation into the perioperative management of RAAS inhibitors and the complex relationship between chronic cardiovascular therapy and surgical outcomes following shoulder arthroplasty.

## Supplementary Information

Below is the link to the electronic supplementary material.

Supplementary Material 1

## Data Availability

All data used in this study were obtained from the TriNetX research network. The data are derived from de-identified electronic health records contributed by participating healthcare organizations and are accessible to authorized users through the TriNetX platform. Access to the underlying data is subject to TriNetX data-use agreements and institutional access permissions.
