# Supplementary material for: Perioperative antihypertensive medication use is associated with no antifibrotic benefit but higher complications and revision rates following shoulder arthroplasty
Source: Eur J Orthop Surg Traumatol. 2026 Aug 1;36(1):314. doi: 10.1007/s00590-026-04883-y (PMC13428774; doi:10.1007/s00590-026-04883-y)
Supplement: Supplementary file 1 — Supplementary Material 1 [file 590_2026_4883_MOESM1_ESM.docx]

Supplementary Table: ICD-10 and CPT Codes Used in the Database

| **Codes** | |
| --- | --- |
|  |  |
| **Shoulder Arthroplasty** | 23472, Z96.61 |
| **ARBs** | 52175, 83515, 1091643, 321064, 73494, 83818, 69749, 214354, 3827 |
| **ACEis** | 1998, 39990, 60245, 30131, 18867, 50166, 38454, 21102, 35208, 35296, 54552, 29046 |
|  |  |
| **Outcomes** |  |
| **90-day** |  |
| ED Visits | Visit: emergency |
| Readmission | Visit: inpatient encounter |
| MI | I21 |
| PE | I26 |
| DVT | I82 |
| Transfusion | 36430 |
| Renal Failure | N19, N17 |
| Sepsis | A41.9, A41.89 |
|  |  |
| **1-yr** |  |
| Capsular Release | 29825 |
| MUA | 23700 |
| Revision | 23473, 23474, 1021145, 609157009 |
| PJI | T84.5 |
| Loosening | T84.03 |
| Dislocation | T84.02 |
